# Bioremediation of High-Concentration Heavy Metal-Contaminated Soil by Combined Use of *Acidithiobacillus ferrooxidans* and Fe_3_O_4_–GO Anodes

**DOI:** 10.3390/toxics13110959

**Published:** 2025-11-06

**Authors:** Alifeila Yilahamu, Xuewen Wu, Xiaonuan Wang, Shengjuan Peng, Weihua Gu

**Affiliations:** School of Resources and Environmental Engineering, Shanghai Polytechnic University, Shanghai 201209, China; 20231516001@sspu.edu.cn (A.Y.); 20221516057@sspu.edu.cn (X.W.); xnwang@sspu.edu.cn (X.W.); sjpeng@sspu.edu.cn (S.P.)

**Keywords:** soil PTE contamination, *Acidithiobacillus ferrooxidans*, graphene oxide, modified anode, bioelectrochemical system

## Abstract

Soils heavily contaminated with potentially toxic elements (PTEs) pose substantial risks to the environment and human health. However, conventional remediation methods are often plagued by high energy consumption and the potential for secondary pollution. To address this challenge, this study developed a synergistic system combining acidophilic bacteria with a Fe-modified anode, aiming to enhance the remediation of PTEs in such contaminated soils. This system integrates the following three core components: the catalytic function of Fe_3_O_4_–graphene-oxide (Fe_3_O_4_–GO) nanocomposites, the acclimation of microbial communities, and the optimization of process parameters—specifically, applied electric current, pH, and oxidation–reduction potential (ORP). Experimental treatments were designed to assess the individual and combined effects of three key factors: bacterial inoculation, the Fe-modified anode, and the addition of Fe_3_O_4_–GO. The results revealed that the integrated synergistic system effectively reduced the soil pH from 2.9 to 2.0 and maintained the ORP at approximately 600 mV. For PTE removal, the system achieved efficiencies of 89% for Zn, 85.89% for Cu, 66.3% for Pb, 77.89% for Cd, and 40.63% for Cr, respectively. In contrast, control groups lacking bacteria, applied current, or Fe_3_O_4_–GO exhibited significantly lower metal removal efficiencies. Notably, the bacteria-free treatment led to a more than 50% reduction in Cr removal. Additionally, the group with an unmodified anode only achieved 1/3 to 1/2 of the removal efficiencies observed in the full synergistic system; this discrepancy is likely attributed to reduced electron transfer efficiency and compromised microbial adhesion on the anode surface. These findings demonstrate that the coupling of electrochemical enhancement, acidophilic microbial activity, and Fe_3_O_4_–GO catalysis constitutes an effective and energy-efficient approach for remediating soils contaminated with high concentrations of PTEs while simultaneously minimizing the risk of secondary pollution.

## 1. Introduction

With the rapid development of industrialization and urbanization, soil pollution has intensified and evolved into a global environmental crisis. In China, the issue of soil environmental safety has attracted great concern, as PTE contamination not only causes ecosystem degradation and biodiversity loss but also poses significant risks to human health through environmental exposure and the food chain [1]. PTE contamination—such as that from chromium (Cr), cadmium (Cd), and lead (Pb)poses severe risks to ecosystems and public health, primarily due to the high toxicity, persistence, and strong bioaccumulation tendency of these metals.

Current soil remediation technologies are generally categorized into physical, chemical, and biological methods. Physical methods are often costly and may cause secondary pollution, whereas chemical methods can disrupt soil structure and introduce new contaminants [2]. Bioremediation is considered more cost-effective and environmentally benign but faces challenges such as long treatment cycles and limited efficacy for pollutants with low bioavailability. Among biological approaches, microbial remediation shows unique advantages in treating organic contaminants and PTEs, though its efficiency remains inadequate when applied independently [3]. Therefore, integrated strategies that combine microbial remediation with other technologies are increasingly being explored [4].

Bioelectrochemical systems (BESs) represent an innovative technology that couples microbial metabolism with electrochemical reactions, effectively overcoming the limitations of conventional remediation approaches [5]. Electrochemical technologies have also attracted increasing attention due to their effectiveness in pollutant removal under various electrode materials and configurations [6]. In BESs, electroactive microorganisms facilitate extracellular electron transfer (EET), oxidizing organic pollutants at the anode while promoting the removal of heavy metals through cathodic reduction. With the dual advantages of low energy consumption and high efficiency, BESs offer a promising alternative to traditional physicochemical and biological methods [7].

Electrode materials are critical to BES performance. Research has shifted from single-component electrodes to high-performance composites [8]. For instance, Song et al. [9] compared graphite felt and activated carbon felt in sediment microbial fuel cells (SMFCs). For example, Chen et al. [10] designed an electrode with reduced internal resistance for microbial fuel cells (MFCs), achieving a power density of 2966 mW/m^2^. Modifications with metal ions/oxides, such as those by Lowy et al. [11], increased current density by 1.5–2.2 times. Similarly, Fe/Fe_2_O_3_-modified anodes achieved substantially higher power densities [12,13], and Fe_3_O_4_ nanoparticles were shown to accelerate electron transfer [14]. FeS_2_-modified anodes also demonstrated exceptional performance, attributed to enriched Geocacher and electroactive biofilm formation [15]. Heteroatom doping (e.g., N, Fe, S) further optimizes EET by enhancing bacterial adhesion and creating electroactive sites [16,17,18]. For example, nitrogen-doped carbon nanotube/graphene composites improved flavin adsorption and biofilm attachment [18], and Fe@Fe_2_O_3_/NCQD hybrids enhanced anode conductivity [19].

As the electroactive component of BESs, microorganisms mediate extracellular electron transfer (EET) that couples anodic oxidation and cathodic reduction, thereby linking microbial metabolism to contaminant transformations [20,21,22]. Since early work demonstrating the microbial use of solid-phase metals as electron acceptors [23], a variety of electroactive strains have been identified and strategies to promote microbe–electrode interactions (e.g., by increasing surface area and promoting biofilm formation) have been developed [24,25,26,27].

BESs possess the unique advantage of enabling simultaneous pollution remediation and energy recovery; however, their effectiveness is constrained by the low mobility of PTEs [28]. The electron transfer kinetics and catalytic properties of electrode materials are decisive for system performance [29]. Fe/C materials efficiently reduce Cr(VI) via micro-electrolysis-generated radicals [11], While advanced adsorbents like metal–organic frameworks (MOFs) demonstrate exceptional heavy metal removal capacities primarily through high surface area adsorption [30]. However, single-mechanism strategies such as pure adsorption or reduction face inherent limitations in complex environments. There is an urgent need to develop integrated, multi-mechanism approaches [31,32,33]. In recent years, comprehensive frameworks for heavy metal pollution management have been established in various regions. China implemented the Soil Pollution Prevention and Control Law and a risk-based management system for agricultural and development land [34]. In Europe, Zlati et al. (2023) analyzed spatio-temporal dynamics of Cd, Pb, and Hg pollution, proposing a policy evolution model to combat HM contamination in water bodies [35]. These efforts reflect global recognition of BESs as a sustainable, low-energy, and efficient platform for heavy metal recovery, supporting their further development and potential large-scale application [36].

Therefore, this study focuses on the remediation of soils collected from an e-waste dismantling area in Taizhou City, Zhejiang Province, China—one of the most severely contaminated sites with high levels of heavy metals, including Zn, Cu, Pb, Cd, and Cr. To address this environmental challenge, we propose a bioelectrochemical system that couples *Acidithiobacillus ferrooxidans* metabolism with an Fe_3_O_4_–graphene oxide (Fe_3_O_4_–GO)-modified anode. This integrated system aims to enhance the dissolution, transformation, and immobilization of heavy metals through a tripartite synergy of biological metabolism, electrochemical regulation, and nanomaterial catalysis. The outcomes of this study provide a green, energy-efficient approach for the remediation of heavy metal-contaminated soils, contributing to the global effort toward sustainable environmental management.

## 2. Materials and Methods

### 2.1. Experimental Materials

The main experimental materials used in this study were as follows: Graphene oxide (GO) was purchased from Suzhou Carbonfeng Graphene Technology Co., Ltd. (Suzhou, China); magnetite (Fe_3_O_4_) from Shenzhen Suiheng Technology Co., Ltd. (Shenzhen, China); graphite rods (GRs) from Beijing Jingke Scientific Instruments Co., Ltd. (Beijing, China); and 5% Nafion solution from DuPont (Wilmington, DE, USA). Other chemical reagents, including (NH_4_)_2_SO_4_, KCl, K_2_HPO_4_, MgSO_4_·7H_2_O, Ca(NO_3_)_2_, and FeSO_4_·7H_2_O, were purchased from Sinopharm Chemical Reagent Co., Ltd. (Shanghai, China).

Soil samples were collected from an e-waste dismantling site in Taizhou City, Zhejiang Province. This area has a long history of informal e-waste recycling, involving manual disassembly and open burning of circuit boards and cables, which has resulted in severe accumulation of Zn, Cu, Pb, Cd, and Cr in the surrounding soils. The sampling procedure strictly followed the Chinese national standard Technical Specification for Soil Environmental Monitoring [37]. A grid method was adopted for arranging sampling points, and non-metallic tools (e.g., plastic shovels) were used to avoid secondary contamination from metal utensils. During sampling, gravel, plant residues, and other debris were carefully removed to ensure sample homogeneity. At each sampling point, 5–10 sub-samples were collected using the “five-point method” (1 sub-sample from the center and 4 from the vertices of a 1 m × 1 m square), which were then thoroughly mixed to form a composite sample.

Composite samples were transported to the laboratory and air-dried in a cool, ventilated environment (25 ± 2 °C) for 5–7 days. The samples were turned regularly during drying to prevent mold growth and ensure uniform moisture loss. After air-drying, residual debris was further removed, and the samples were ground using an agate mortar until they passed through a 2 mm nylon sieve. Representative samples (~500 g each) were obtained via the “quartering method.” These processed samples were stored in polyethylene bags at room temperature, with protection from light and moisture, until subsequent analysis.

The bacterial strain used in this study, *A. ferrooxidans*, was isolated through strict screening, repeated enrichment, and acclimatization from soil samples collected from the Dexing Copper Mining Area, Jiangxi Province. *A. ferrooxidans* cultures were incubated in a constant-temperature shaking incubator at a rotation speed of 130 rpm, following established microbiological techniques.

### 2.2. Synthesis and Characterization of Fe_3_O_4_–GO Anode

#### 2.2.1. Preparation for Composite Material

**Figure 1:**
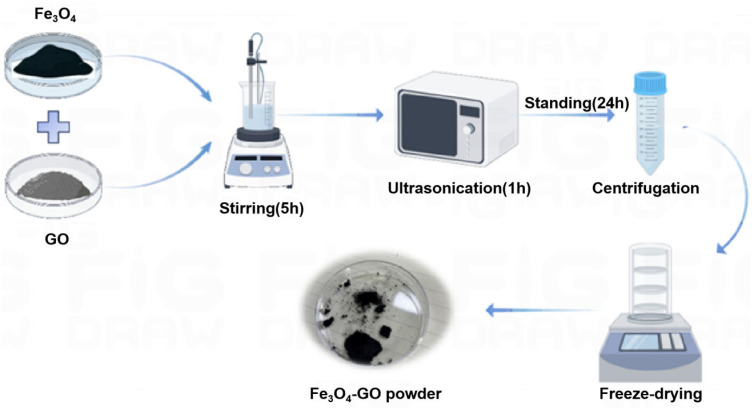
Material preparation diagram.

Leveraging the distinct surface charge properties of Fe_3_O_4_ (positively charged) and graphene oxide (GO, negatively charged), this study adopted an electrostatic self-assembly method to fabricate the Fe_3_O_4_–GO composite material. The detailed preparation procedure was as follows: (1) 50 mg of GO was dispersed in 50 mL of deionized water. A homogeneous GO suspension was obtained after 5 h of mechanical stirring and 1 h of ultrasonication; (2) 1 g of Fe_3_O_4_ was added to the above GO suspension, and the dispersion process (mechanical stirring followed by ultrasonication) described in Step 1 was repeated to ensure uniform mixing of Fe_3_O_4_ and GO; (3) After the mixture was statically placed for 24 h, it was separated via centrifugation. The collected precipitate was washed twice with ethanol and then air-dried; (4) The air-dried sample was pre-frozen for 4 h, followed by freeze-drying (lyophilization) for 24 h to obtain the Fe_3_O_4_–GO composite. The final Fe_3_O_4_–GO product was stored at 4 °C for subsequent use (the preparation process is illustrated in Figure 1).

#### 2.2.2. Electrode Preparation

The modified anode was fabricated using a dip-coating method developed for this study. First, the Fe_3_O_4_–GO active material was dispersed in ethanol, and Nafion was added at a mass ratio of 1:5 (active material: Nafion). The mixture was then sonicated for 1 h to form a homogeneous suspension.

Next, the pre-treated graphite rod was immersed in this suspension for 1 h and subsequently air-dried. This dip-coating–air-drying cycle was repeated three times to ensure uniform deposition of the active material on the graphite rod surface. After completing the cycles, the suspension was precisely pipetted onto the active area of the electrode using a micropipette, ensuring consistent surface coverage across the electrode. Finally, the electrode was cured at room temperature to evaporate the solvent, resulting in a fully functionalized Fe_3_O_4_–GO-modified anode.

#### 2.2.3. Structural Characterization of Fe_3_O_4_–GO Composite Material

The crystalline structure of the samples was analyzed by X-ray diffraction (XRD, D8 Advance, Bruker, Karlsruhe, Germany). The functional groups were determined by Fourier-transform infrared spectroscopy (FT-IR, Nicolet iS10, Thermo Fisher Scientific, Waltham, MA, USA). The surface area and pore size distribution were measured using a Brunauer–Emmett–Teller analyzer (BET, ASAP 2460, Micromeritics, Norcross, GA, USA). The morphology and microstructure were observed by scanning electron microscopy (SEM, SU8010, Hitachi, Tokyo, Japan). The pH and ORP were monitored using a Multi-Parameter Meter (HQ40D, Hach, Loveland, GO, USA), and the metal concentrations were analyzed by inductively coupled plasma optical emission spectrometry (ICP-OES, Optima 8000, PerkinElmer, Waltham, MA, USA). Data plotting and further data processing were performed using Origin 2018 (OriginLab, https://www.originlab.com/).

### 2.3. Construction of BP-C-MEDC

The remediation system was established using a custom 150 mL electrolytic cell, comprising a Fe_3_O_4_–GO-modified anode (Φ6 × 90 mm) and a graphite cathode, filled with 90 mL of pH 2.0 9K medium (per liter: 3.0 g (NH_4_)_2_SO_4_, 0.1 g KCl, 0.5 g K_2_HPO_4_, 0.5 g MgSO_4_·7H_2_O, 0.01 g Ca(NO_3_)_2_, and FeSO_4_·7H_2_O as energy source) containing 44.2 g/L FeSO_4_·7H_2_O and 1 g of contaminated soil. Following inoculation with 10% *A. ferrooxidans* bacterial suspension, finally, the electrodes were inserted and connected to a constant-current power supply set at 20 mA (0.7 mA/cm^2^). The system was operated at 30 °C for 7 d.

Five control groups were designed by varying the presence of modified anode, inoculation, applied current, and Fe_3_O_4_–GO (Table 1). With Group 1 (Fe_3_O_4_–GO-modified anode + inoculation + soil + current) as the full experimental group, single-variable control was achieved by excluding inoculation (Group 2) to verify *A. ferrooxidans*’ role; omitting Fe_3_O_4_–GO (Group 3) to test Fe_3_O_4_–GO’s effect; removing soil (Group 4) to eliminate non-soil interference on PTE detection; and replacing the modified anode with pure graphite (Group 5) to confirm the anode modification’s contribution—ensuring reliable experimental conclusions.

After 7 d, total PTE concentrations in soil (pre- and post-remediation) were determined to calculate removal efficiency. Soil samples were digested according to the Microwave Digestion Method for Total Metal Elements in Soil and Sediment [38] and analyzed using an inductively coupled plasma optical emission spectrometer (ICP-OES, Thermo Scientific iCAP 7200, Waltham, MA, USA).

Removal Rate Calculation: The removal rate of PTEs was calculated using the standard formula:

$$Removal\;Rate\left(\%\right)=\left[\frac{C_{0}-C_{t}}{C_{0}}\times C_{0}\right]\times 100\%$$

where: C_0_ = initial concentration of PTEs in soil (mg/kg); C_t_ = concentration of PTEs in soil after remediation (mg/kg).

Additionally, the following quality control and operational details were implemented:

PTE analysis: After filtration, the digested solution was stored at 4 °C for subsequent ICP-OES detection. All operations were conducted in a fume hood with acid-resistant gloves and goggles, using deionized water and ultra-pure reagents to avoid cross-contamination.

pH measurement: pH of the culture medium was measured daily with a pH meter, which was calibrated with standard buffer solutions (pH 4.00, 6.86, 9.18) before use, with 3 replicates set for each treatment.

ORP measurement: ORP values were determined daily using an oxidation–reduction potential meter (STARTER3100, Shanghai Ohaus Instruments Co., Ltd., Shanghai, China) simultaneously with pH. A bacteria-free control group was included to eliminate abiotic interference.

**Figure 2:**
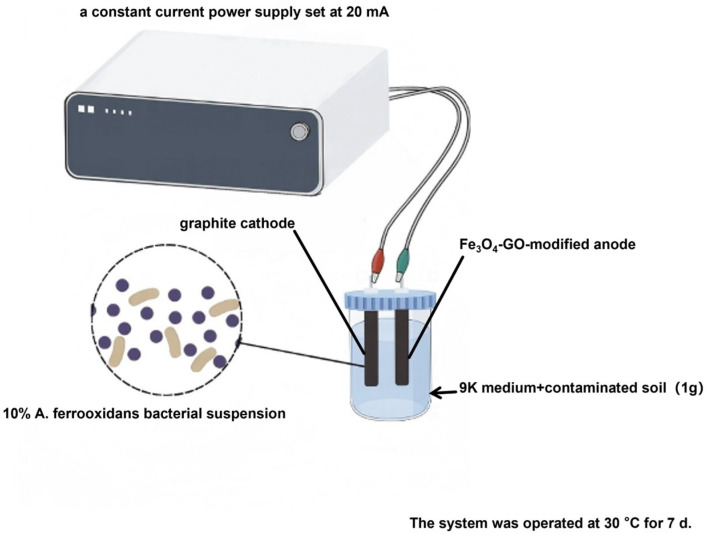
Schematic diagram of BES structure and optimization of PTE removal performance parameters.

All experiments were performed in triplicate, and data are presented as mean ± standard deviation (SD). Statistical significance among the five treatment groups (Groups 1–5) was analyzed using one-way analysis of variance (ANOVA) followed by Tukey’s post hoc test (*p* < 0.05) in SPSS 26.0 (IBM, Armonk, NY, USA). The BES structure and optimization of PTE removal parameters are illustrated in Figure 2.

## 3. Results and Discussion

### 3.1. Performance of the Fe_3_O_4_–GO Anode

#### 3.1.1. Structural Properties of Fe_3_O_4_–GO Composite Material

**Figure 3:**
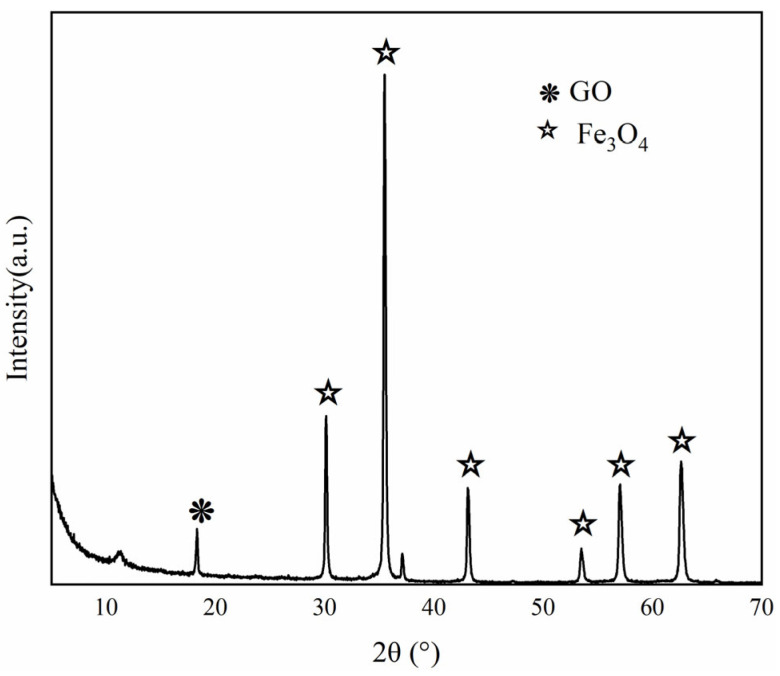
X-ray diffractogram of the composite (Fe_3_O_4_–GO) (symbols represent the characteristic peaks of both, respectively).

The X-ray diffraction (XRD) pattern of the Fe_3_O_4_–GO composite is presented in Figure 3. A characteristic broad and low-intensity peak for GO is observed at approximately 10°, indicating a large interlayer spacing within its hexagonal lattice, attributable to oxygen-containing functional groups (e.g., hydroxyl, carboxyl) on its surface. For the Fe_3_O_4_ component, sharp, high-intensity diffraction peaks are detected at 2θ values of 30.1° ((220)), 35.6° ((311)), 43.3° ((400)), 53.5° ((422)), 57.1° ((511)), and 62.6° ((440)). These peaks are consistent with the standard spinel crystal structure of Fe_3_O_4_ (JCPDS No. 19-0629), confirming the preservation of its crystallinity after composite formation with GO.

**Figure 4:**
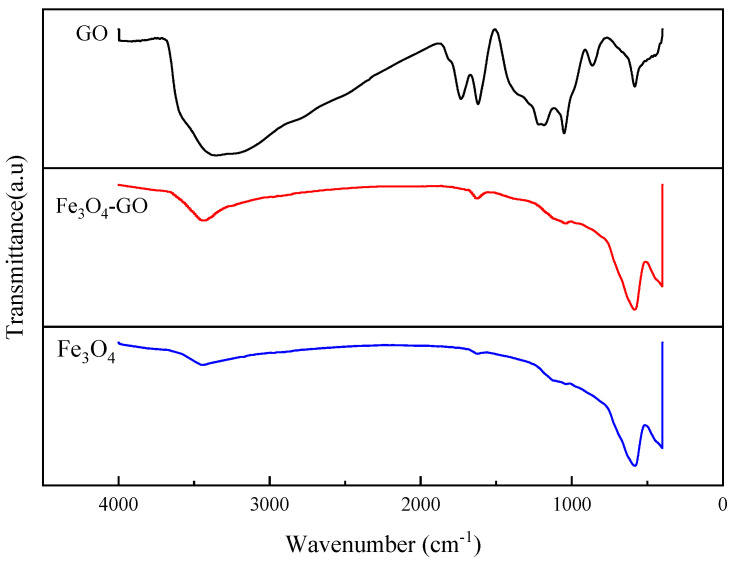
FT-IR comparison of GO, Fe_3_O_4_ and Fe_3_O_4_–GO.

Fourier-transform infrared (FT-IR) spectroscopy was employed to further investigate the chemical structure and interactions (Figure 4). The spectrum of pure GO exhibits distinct absorption peaks at: 3420 cm^−1^ (O–H stretching vibration), 1725 cm^−1^ (C=O stretching from carboxyl groups), 1627 cm^−1^ (C=C stretching from the graphene skeleton), 1392 cm^−1^ (O–H deformation vibration), and 1224/1050 cm^−1^ (C–O–C vibrations). In the spectrum of the Fe_3_O_4_–GO composite, characteristic peaks are observed at 3425 cm^−1^ (O–H stretching), 1633 cm^−1^ (C=C stretching), 1399 cm^−1^ (COO^−^ stretching), 1113 cm^−1^ (C–O stretching), and notably, at 590 cm^−1^ (Fe–O stretching vibration from Fe_3_O_4_), confirming the successful incorporation of Fe_3_O_4_. The retention of characteristic peaks from both components, coupled with observable shifts in peak positions (e.g., O–H from 3420 to 3425 cm^−1^) and variations in intensity (e.g., reduction of the C=O peak), provides strong evidence for interfacial interactions, likely via chemical bonding such as coordination between Fe ions and oxygen-containing functional groups. These findings are consistent with previous reports on component interactions in similar composite systems [39,40].

**Figure 5:**
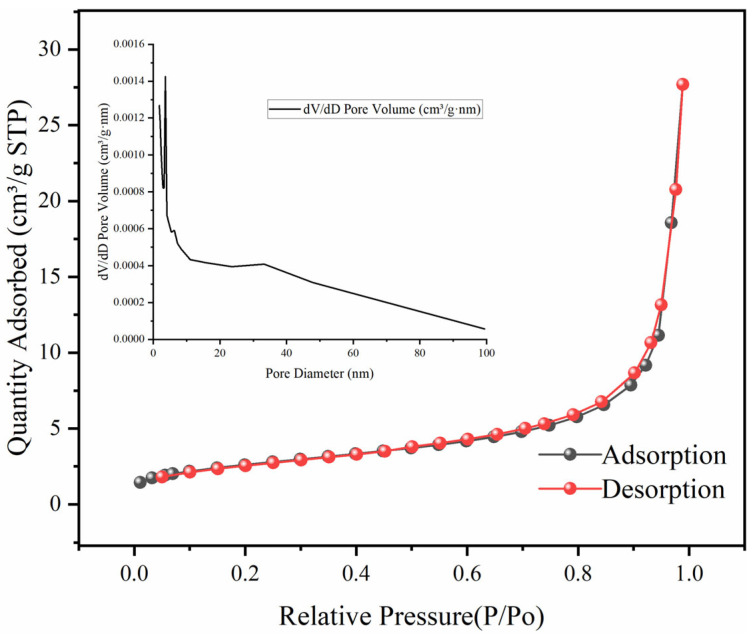
BET plot of Fe_3_O_4_–GO.

The porous structure of the Fe_3_O_4_–GO composite was analyzed by N_2_ adsorption-desorption measurements (Figure 5). The isotherm exhibits a typical Type IV curve with an H3-type hysteresis loop, characteristic of mesoporous materials with slit-like pores, aligning with the layered morphology of GO. A significant increase in adsorption capacity was observed within the relative pressure (P/P_0_) range of 0.4–0.9. The desorption branch forms a distinct hysteresis loop initiating at P/P_0_ ≈ 0.45, confirming a pore size distribution primarily concentrated in the mesoporous range (2–10 nm), with a dominant peak centered at 3.8 nm.

This hierarchical porous structure originates from the uniform dispersion of Fe_3_O_4_ nanoparticles between the GO layers. The Fe_3_O_4_ nanoparticles act as effective “spacers,” physically separating adjacent GO sheets and thereby inhibiting their restacking—a common issue that reduces the accessible surface area in pristine GO. Consequently, the Fe_3_O_4_–GO composite demonstrates significantly enhanced specific surface area and a high mesopore volume.

Scanning electron microscopy (SEM) analysis (Figure S1) confirmed the successful formation of the Fe_3_O_4_–GO composite. Fe_3_O_4_ nanoparticles were uniformly distributed across the GO sheets, forming a continuous and porous network without visible phase separation. This structural arrangement ensures strong interfacial contact between Fe_3_O_4_ and GO, consistent with the BET results.

The hierarchical and conductive framework of the Fe_3_O_4_–GO composite provides efficient mass and charge transport pathways. These features are expected to enhance anode capacitive behavior and electron transfer efficiency, thereby improving the overall electrochemical performance of the BES.

#### 3.1.2. Electrochemical Performance of the Fe_3_O_4_–GO Anode

Cyclic voltammetry (CV) measurements were performed to evaluate the electrochemical performance of the prepared electrodes. A standard three-electrode system was employed on an Interface 1010B electrochemical workstation, with the following configuration:

Working electrode: The Fe_3_O_4_–GO-modified graphite rod (or unmodified graphite rod, for comparison).

Counter electrode: A platinum wire (0.5 mm diameter, 99.99% purity);

Reference electrode: A Ag/AgCl electrode (3 M KCl, model RE-1B).

The electrolyte used was a 50 mM phosphate buffer solution (PBS, pH 7.0) supplemented with 0.1 M NaCl. Prior to measurements, the electrolyte was purged with high-purity nitrogen for 30 min to eliminate dissolved oxygen, which could interfere with redox reactions. CV scans were conducted at a constant temperature of 25 ± 1 °C, over a potential range of −0.8 to 0.8 V (vs. Ag/AgCl), and at a scan rate of 10 mV/s. To minimize the influence of double-layer charging effects, three consecutive scans were recorded at 0.001 V intervals. After baseline correction using Origin 2022 software, the third scan (which exhibited stable electrochemical behavior) was selected for subsequent analysis.

**Figure 6:**
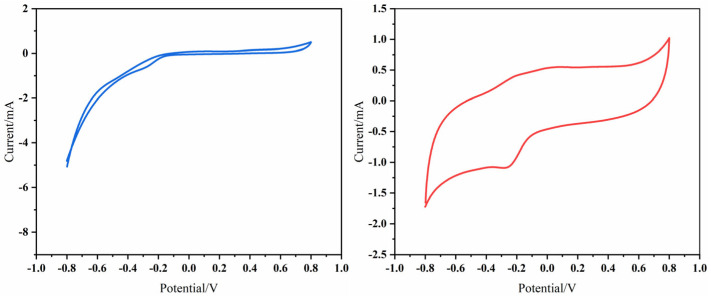
Cyclic voltammetry curves of unmodified anode vs. modified anode at 10 mv/s.

As presented in Figure 6, the CV curves obtained at 10 mV/s reveal distinct differences in electrochemical behavior between the unmodified graphite anode and the Fe_3_O_4_–GO-modified anode. The unmodified electrode exhibits a smooth CV profile with weak, ill-defined redox peaks and a maximum peak current density of only 0.28 mA/cm^2^. This observation indicates that the unmodified anode has limited electrochemically active sites and suffers from slow electron transfer kinetics—key factors restricting its performance.

In sharp contrast, the Fe_3_O_4_–GO-modified anode displays well-resolved redox peaks centered at approximately −0.2 V and 0.3 V (vs. Ag/AgCl), accompanied by a significant enhancement in current response. This improvement, coupled with an increased electrochemically active surface area (derived from the hierarchical porous structure of Fe_3_O_4_–GO), reflects three critical advantages of the modified anode: accelerated redox reaction kinetics, superior electrochemical activity, and enhanced current generation capacity. These results directly confirm the effectiveness of Fe_3_O_4_–GO modification in boosting the electrochemical performance of the anode.

### 3.2. Removal Efficiency for PTEs in Soil by Acclimated Strains

Analysis of PTE removal efficiency across successive bacterial generations revealed a pronounced upward trend in the removal of Zn, Cu, Pb, Cd, and Cr as the number of acclimation cycles increased (Figure S2). After four acclimation generations, the removal efficiency of Zn increased from an initial 40% to 66%, Cu from 28% to 50%, and Cd from 20% to 65%. In contrast, Pb and Cr exhibited more modest improvements, with their removal efficiencies reaching 45% and 38%, respectively, after the same period.

Notably, the removal efficiency of Cu plateaued between the 2nd and 3rd generations, with only a 6% increase observed during this interval. This plateau is likely attributed to the inhibition of bacterial metal-transport genes under elevated Cu^2+^ concentrations—high levels of Cu^2+^ may disrupt the expression or function of genes encoding transporters responsible for Cu^2+^ uptake and sequestration, thereby limiting further improvements in removal efficiency. Additionally, the relatively low removal efficiency of Cr (VI) (maximizing at 38%) is associated with its unique remediation mechanism: Cr (VI) requires reduction to the less toxic and more easily immobilized Cr (III) before effective removal, and this reduction step is constrained by the activity of bacterial reductases (e.g., cytochrome c reductase) in the system.

Concurrent monitoring of pH and oxidation–reduction potential (ORP) during the acclimation process showed that the culture medium pH decreased from an initial 3.1 to 2.2. The resulting acidic environment likely exerted dual effects on PTE removal: it facilitated the dissolution of Zn^2+^ and Cd^2+^ from soil matrices (increasing their bioavailability for bacterial uptake) and enhanced their adsorption onto the Fe_3_O_4_–GO-modified anode, while simultaneously constraining Cr(VI) reduction—acidic conditions can suppress the activity of reductase enzymes critical for Cr(VI) → Cr(III) conversion.

Overall, these findings demonstrate that multi-generational acclimation enhances two key microbial traits: tolerance to high PTE concentrations and metabolic adaptation to the contaminated environment. Together, these improvements significantly boost the microbial community’s capacity for PTE removal.

### 3.3. System-Level Remediation Efficiency

#### 3.3.1. Changes in System pH and ORP

**Figure 7:**
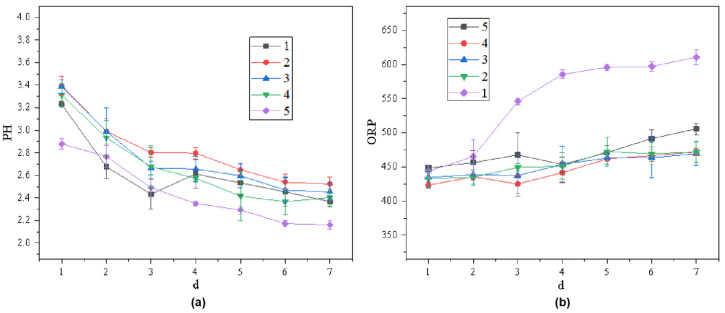
Variations in (**a**) pH and (**b**) ORP during remediation under different treatment groups.

Variations in system pH and oxidation–reduction potential (ORP) during the remediation operation are shown in Figure 7. Among all experimental groups, Group 5 (modified anode + Fe_3_O_4_–GO + *A. ferrooxidans* + applied current) exhibited the most favorable conditions, with pH decreasing from 2.9 to ≈2.0 and ORP stabilizing near 600 mV.

These trends reflect the synergistic effects of three concurrent processes: (i) electric field-driven H^+^ migration caused by anodic water electrolysis, (ii) sustained Fe^3+^/Fe^2+^ redox cycling mediated by *A. ferrooxidans*, and (iii) Fe_3_O_4_–GO–induced micro-electrolysis that accelerates electron transfer and metal ion transport. The conductive GO matrix also enhances H^+^ distribution and facilitates PTE adsorption, further improving redox efficiency.

In contrast, control groups lacking any of the key components (bacterial inoculation, Fe_3_O_4_–GO, applied current, or anode modification) showed slower pH decline and unstable ORP profiles. These results confirm that the coupling of electrochemical stimulation, Fe_3_O_4_–GO modification, and microbial oxidation is essential for the superior redox performance of the integrated system.

Groups 1–5 correspond to:

1—modified anode + bacteria + electricity + Fe_3_O_4_–GO.

2—modified anode + electricity + Fe_3_O_4_–GO.

3—modified anode + bacteria + electricity.

4—modified anode + bacteria + Fe_3_O_4_–GO.

5—unmodified anode + bacteria + electricity + Fe_3_O_4_–GO.

“d” represents days, and ORP is expressed in mV.

#### 3.3.2. Effect of Fe_3_O_4_–GO on PTE Removal Efficiency in the System

**Figure 8:**
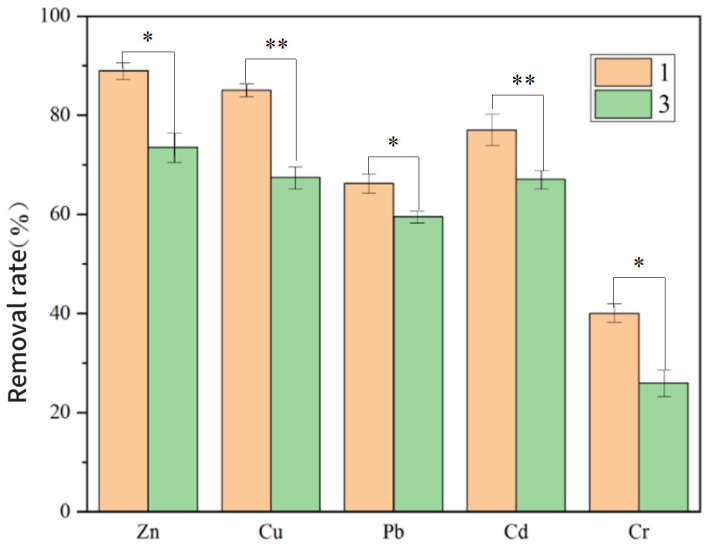
Comparison of PTE removal rate in soil of group 1 and group 3 (*p* < 0.05 *, *p* < 0.01 **).

As shown in Figure 8, the Fe_3_O_4_–GO treatment group achieved markedly higher removal efficiencies for all PTEs than the control group without Fe_3_O_4_–GO (Group 3). This improvement primarily arises from three synergistic mechanisms: (i) the Fe_3_O_4_–GO composite provides abundant adsorption sites through its high surface area and oxygen-containing functional groups (–OH, –COOH); (ii) these functional groups interact with the extracellular polymeric substances (EPSs) secreted by *A. ferrooxidans*, forming a stable bio–nano interface that enhances metal ion adsorption and microbial reduction; and (iii) the magnetic Fe_3_O_4_ structure facilitates biomass retention and recovery, maintaining continuous microbial activity and preventing secondary metal release.

As a result of these coupled effects—physical/chemical adsorption (Fe_3_O_4_–GO), biological reduction (microbial activity), and electrochemical deposition (electrode reactions)—the Fe_3_O_4_–GO group exhibited faster and more stable removal kinetics than the non- Fe_3_O_4_–GO group, which relied solely on microbial adsorption and electrode deposition.

Supplementary Figure S3 further confirms that the enhancement was most pronounced for Pb and Cd, while moderate improvements were observed for Zn and Cu. The relatively weaker response of Zn^2+^ and Cu^2+^ can be attributed to their higher solubility and weaker complexation under acidic conditions. Nevertheless, Fe_3_O_4_–GO still promoted their adsorption and co-precipitation via surface hydroxyl and carboxyl groups, indicating a broad enhancement effect on multi-metal removal.

In conclusion, the introduction of Fe_3_O_4_–GO effectively optimizes the removal dynamics of multiple PTEs by enhancing both initial adsorption capacity and long-term remediation sustainability.

#### 3.3.3. Effect of Electric Current on PTE Removal Efficiency in the System

**Figure 9:**
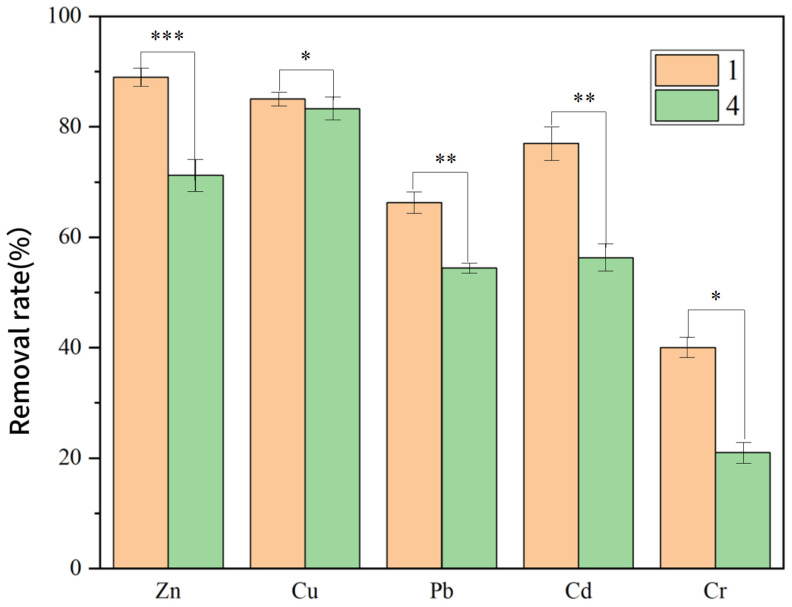
Comparison of PTE Removal Rates in Soil between Group 1 and Group 4. (*p* < 0.05 *, *p* < 0.01 **, *p* < 0.001 ***).

Figure 9 demonstrates significantly higher removal efficiencies for all five target PTEs (Zn, Cu, Pb, Cd, and Cr) in the electrified group (Group 4) compared to the non-electrified control (Group 1). The electrified group achieved final removal rates of 89% (Zn), 85.9% (Cu), 66.3% (Pb), 77.9% (Cd), and 40.6% (Cr), representing an average improvement of approximately 14.8 percentage points.

The applied electric field triggered a cascade of electrochemical and biological effects. The Fe_3_O_4_–GO anode formed a strong electric double layer, intensifying H^+^ migration and promoting Fe^2+^ release from the composite surface, which enhanced metal-ion transport and electrodeposition. At the same time, the field-maintained Fe^3+^/Fe^2+^ redox cycling and stimulated the metabolic activity of *A. ferrooxidans*, jointly strengthening bioreduction of multivalent metals such as Cr (VI).

As illustrated in Figure S4, the electrified system exhibited faster removal kinetics throughout the remediation process. Zn and Cu showed rapid initial declines within the first three days, whereas Pb, Cd, and Cr maintained larger efficiency gaps compared with the control, with final differences of 11.9%, 20.8%, and 19%, respectively.

These results demonstrate a robust tripartite synergy—electrochemical driving + nanomaterial catalysis + biotransformation—that accelerates ion transport, promotes electrodeposition, and sustains microbial reduction, collectively enabling efficient and sustained PTE removal.

#### 3.3.4. Effect of Modified Electrode on PTE Removal Efficiency in the System

**Figure 10:**
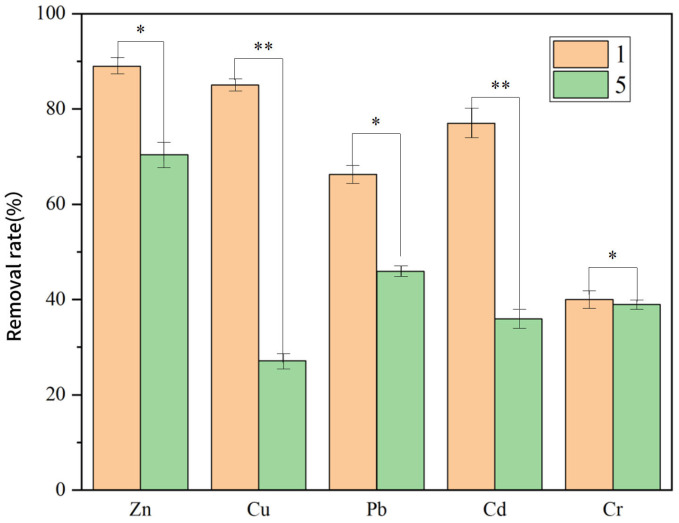
Comparison of PTE Removal Rates in Soil between Group 1 and Group 5. (*p* < 0.05 *, *p* < 0.01 **).

Figure 10 compares the removal efficiencies of the five target PTEs (Zn, Cu, Pb, Cd, and Cr) between Group 1 (unmodified anode group) and Group 5 (Fe_3_O_4_–GO-modified anode group). The results show that the modified anode group achieved significantly higher removal efficiencies for all five PTEs than the unmodified anode group—clearly indicating that anode modification effectively enhances the overall performance of the microbial–electrochemical remediation system.

Although the unmodified anode group generally exhibited lower removal efficiency, it still retained a certain degree of PTE removal capacity. This confirms that the unmodified anode maintains fundamental functionality under microbial–electrochemical synergy (i.e., the collaboration between microorganisms and electrochemical reactions). This observation aligns with the findings of Zheng et al. [41], where immobilized bacterial strains achieved higher ammonia nitrogen removal efficiency than free bacteria. Their study indirectly supports that microbial activity alone can contribute partially to pollutant removal—even in the absence of optimized electrode materials.

For the modified anode (modified with SiO_2_ and chitosan), the surface functional groups of Fe_3_O_4_ nanoparticles are significantly enriched, which enhances electron transfer efficiency and thereby strengthens the synergistic interaction between microorganisms and the electrode. The specific effects on individual PTEs are as follows:

Zn and Cu: The modified anode group exhibited notably higher removal efficiencies. For Cu in particular, the modified anode likely promotes bioleaching by accelerating electron transfer at the microbe–electrode interface—overcoming the kinetic limitations of Cu^2+^ transformation. In contrast, the unmodified anode group showed lower removal efficiency, which may be attributed to two key factors: reduced reaction kinetics at the microbe–electrode interface, and weaker adsorption capacity and electrochemical synergy compared to the modified anode.

Pb: The superior performance of the modified anode group may stem from its surface characteristics (e.g., increased active sites, enhanced conductivity), which either facilitate the electrodeposition of Pb^2+^ onto the anode surface or promote Pb^2+^ biosorption by attached microorganisms.

Cd: The modified anode potentially accelerates the dissolution of Cd-containing soil minerals and the migration of Cd^2+^ to the electrode by enhancing redox reactions in the system—thereby improving Cd removal.

Cr: The removal efficiency gap between the two groups was minimal. This may be because the anode modification (SiO_2_/chitosan coating) did not effectively facilitate the critical step of Cr (VI) reduction to Cr (III), a prerequisite for reducing Cr toxicity and promoting its precipitation (and thus removal) from the soil.

In summary, the modified anode enhances the system’s electrocatalytic activity, which accelerates the redox reactions of PTEs. Additionally, the modified anode likely possesses higher electrical conductivity and corrosion resistance: the former ensures efficient electron transfer, while the latter extends the system’s operational longevity and maintains stable performance. In contrast, the surface structure of the unmodified anode (e.g., fewer active sites, lower conductivity) may restrict microbial attachment and hinder electron transfer—limiting its remediation capacity.

Supporting data in Figure S5 further indicate that the Fe_3_O_4_–GO-modified anode increased electrode conductivity, promoted the formation of thicker and more electroactive biofilms (≈50% thicker and ≈40% higher electron-transfer efficiency), and suppressed competing side reactions such as oxygen evolution. As a result, the initial removal rate of Group 5 was 2–3 times higher than that of Group 1, and its final removal efficiency also showed substantial improvement.

Overall, anode modification effectively couples microbial metabolism with electrochemical processes, leading to faster electron transport, more stable biofilm activity, and superior multi-metal remediation performance.

#### 3.3.5. Tripartite Synergistic Mechanism of Fe_3_O_4_–GO in Enhancing PTE Removal

The efficient remediation of PTE-contaminated soil by the Fe_3_O_4_–GO-modified anode coupled with *A. ferrooxidans* relies on the tripartite synergy of biological metabolism, electrochemical regulation, and nanomaterial catalysis. These interrelated mechanisms collectively promote the dissolution, transformation, and immobilization of PTEs, which are elaborated in detail as follows.

##### Biological Metabolism-Driven Transformation by *A. ferrooxidans*

As a chemoautotrophic microorganism, *A. ferrooxidans* plays a pivotal role in driving transformations of PTEs speciation through its metabolic activities. Two key metabolic processes underpin this role, as detailed below:

1. Metabolic acidification promotes the dissolution of solid-phase PTEs

During growth and metabolism, *A. ferrooxidans* generates large quantities of H^+^ ions, which reduce the system pH from an initial 2.9 to approximately 2.0. This highly acidic environment disrupts the coordination bonds between PTEs (e.g., Zn, Cd) and soil matrix components—such as clay minerals (e.g., montmorillonite) and soil organic matter (e.g., humic acids). This bond disruption facilitates the conversion of immobile solid-phase PTEs into soluble, bioavailable ions (e.g., Zn^2+^, Cd^2+^), which are more accessible for subsequent removal via adsorption, electrochemical deposition, or bioreduction.

Consistent with previous studies [42], *A. ferrooxidans* induces PTE leaching through two complementary mechanisms: (1) a synergistic “adsorption–secretion–oxidation” process, where the bacterium first adsorbs metal ions onto its cell surface, secretes acidic metabolites (e.g., sulfuric acid) to dissolve surrounding solid-phase metals, and then oxidizes reduced metal species to enhance their solubility; and (2) mineral erosion, where metabolic acids and oxidative byproducts create micro-pores in soil minerals. These micro-pores expose encapsulated or structurally stable PTEs (e.g., metals trapped in mineral lattices), further promoting their release into the aqueous phase.

2. Redox cycling mediates H^+^ production and enhances metal bioavailability

*A. ferrooxidans* also mediates critical Fe–S redox reactions via extracellular electron transfer (EET), which not only sustains its chemoautotrophic metabolism but also contributes to system acidification and changes in metal speciation. To further illustrate these coupled redox processes, representative reactions summarized from previous studies [43,44] are presented in Equations (1)–(7). These reactions describe the oxidation of metal sulfides and ferrous iron mediated by *A. ferrooxidans*, the subsequent formation of ferric hydroxy sulfates, and the generation of protons during these processes:

(1) $$MS+2O_{2}\overset{A.f.}{\to }MSO_{4}$$

(2) $$2FeSO_{4}+0.5O_{2}+H_{2}{SO}_{4}\overset{A.f.}{\to }Fe_{2}{\left(SO_{4}\right)}_{3}+H_{2}O$$

(3) $$4Fe_{2}{\left(SO_{4}\right)}_{3}+2MS+4H_{2}O+2O_{2}\to 2M^{2+}+2{SO_{4}}^{2-}+8FeSO_{4}+4H_{2}SO_{4}\;$$

(4) $$8Fe^{3+}+{SO_{4}}^{2-}+14H_{2}O\to Fe_{8}O_{8}{\left(OH\right)}_{6}SO_{4}+22H^{+}$$

(5) $$3Fe^{3+}+X^{+}+2{HSO_{4}}^{-}+6H_{2}O\to XFe_{3}{\left(SO_{4}\right)}_{2}{\left(OH\right)}_{6}+8H^{+}$$

(6) $$Fe^{3+}+3H_{2}O\to Fe{\left(OH\right)}_{3}+3H^{+}$$

(7) $$H_{2}SO_{4}+materials-M\to materials-2H+MSO_{4}\;$$

These coupled oxidation and hydrolysis reactions explain the continuous decrease in pH and increase in ORP observed in the experiment. The production of Fe^3+^ and H_2_SO_4_ facilitates the transformation of complex-bound potentially toxic elements (PTEs) such as Cu–humic acid and Pb–fulvic acid into free ionic forms (Cu^2+^, Pb^2+^).

In the present experiment, to optimize *A. ferrooxidans*’s metabolic activity, ferrous iron (as a substrate) and 3.0 mL of 5 mol L^−1^ sulfuric acid were added to initiate acidification. Within two days, the system pH stabilized between 1.5 and 1.7, an acidic range that further promotes PTE transformation. As reported in [42], such dissociation significantly increases metal bioavailability, laying the foundation for efficient removal via subsequent microbial–electrochemical processes.

##### Catalysis and Adsorption Mediated by Fe_3_O_4_–GO Nanocomposites

In terms of biofilm promotion, the Fe_3_O_4_–GO composite exhibits a rough surface topography that provides favorable anchoring sites for the attachment and colonization of *A. ferrooxidans*, ultimately facilitating the formation of a dense, continuous biofilm. Scanning electron microscopy (SEM) images (not explicitly referenced herein but consistent with morphological observations in prior sections) confirm that the biofilm coverage on the Fe_3_O_4_–GO-modified anode is significantly higher than that on the unmodified graphite anode. This increased coverage strengthens the microbe–electrode interfacial interaction—an essential prerequisite for efficient extracellular electron transfer (EET) between *A. ferrooxidans* cells and the electrode surface.

Fundamentally, this bio-material synergistic mechanism for enhanced electron transfer relies on two core factors: (1) the catalytic role of electrochemically active *A. ferrooxidans* cells at the anode—these bacteria oxidize organic or inorganic substrates (e.g., Fe^2+^) and transfer the generated electrons to the electrode via EET pathways; and (2) the physicochemical properties of the Fe_3_O_4_–GO composite, which not only support biofilm formation but also reduce the electron transfer resistance at the microbe–electrode interface. As highlighted in previous studies [45], the strength of this microbe–electrode interaction directly dictates the kinetics of PTEs ion transformation (e.g., reduction of Cr (VI) to Cr (III), oxidation of toxic metal species) and, consequently, the overall removal efficiency of PTEs in the microbial-electrochemical system.

To systematically evaluate the individual and synergistic contributions of the key components—electrical stimulation, Fe_3_O_4_–GO nanocomposite, *A. ferrooxidans* inoculation, and anode modification—to the overall remediation performance, a comparative summary of the PTE removal efficiencies across the different experimental groups is presented in Table 2. The results clearly indicate that the integration of all components is essential to achieve the highest remediation efficiency, thereby confirming the proposed tripartite synergy mechanism.

## 4. Conclusions

This study developed a microbial–electrochemical synergistic remediation system using a Fe_3_O_4_–GO (magnetite–graphene oxide)-modified anode. The results show that the synergy among electrical stimulation, Fe_3_O_4_–GO nanocatalysis, and *A. ferrooxidans* metabolism markedly improved the removal of PTEs (Zn, Cu, Pb, Cd, and Cr) from contaminated soil.

Control experiments confirmed the distinct roles of each factor: electrical current accelerated ion migration and electrochemical reactions; Fe_3_O_4_–GO provided abundant adsorption sites and enhanced electron transfer; and *A. ferrooxidans* mediated acidification and bioreduction of metals such as Cr (VI). Together, these effects formed a stable “electrochemical–biological–nanocatalytic” synergy that no single component could achieve alone.

This work demonstrates the feasibility of coupling microbial metabolism with Fe_3_O_4_–GO-modified electrodes for efficient soil remediation. By integrating electrochemical acceleration, nanomaterial catalysis, and microbial transformation, the system overcomes key limitations of conventional methods and prevents secondary metal release through in situ immobilization. Although the tests were conducted under laboratory conditions, future research should focus on pilot-scale validation, scalable electrode design, and energy integration to enable field applications.

## Figures and Tables

**Table 1:** Experimental design and configuration of the five treatment groups.

Serial Number	Electrode (Anode/Cathode)	Inoculation (10%)	Soil (1 g)	Current (20 mA)	Fe_3_O_4_–GO
1	Fe_3_O_4_–GO/GR	√	√	√	√
2	Fe_3_O_4_–GO/GR	×	√	√	√
3	Fe_3_O_4_–GO/GR	√	√	√	×
4	Fe_3_O_4_–GO/GR	√	√	×	√
5	GR/GR	√	√	√	√

**Table 2:** Comparison of the individual and combined contributions of key factors to the remediation efficiency of PTEs.

Experimental Group	Configuration	Zn Removal (%)	Cu Removal (%)	Pb Removal (%)	Cd Removal (%)	Cr Removal (%)
Group 1	Full system	89.0	85.9	66.3	77.9	40.6
Group 2	No Bacteria	76.6	31.6	47.6	38.9	29
Group 3	No Fe_3_O_4_–GO	73.5	67.5	59.5	67.1	26
Group 4	No Current	71.2	83.3	54.4	56.2	21.7
Group 5	Unmodified Anode (Graphite)	70.4	27.2	45.9	35.9	38.1

## Data Availability

The original contributions presented in this study are included in the article/Supplementary Material. Further inquiries can be directed to the corresponding author.

## Supplementary Materials

The following supporting information can be downloaded at: https://www.mdpi.com/article/10.3390/toxics13110959/s1, Table S1: Basic Properties of the Test Soil Sample. Figure S1: Electron microscopy of (a) Fe_3_O_4_, (b) GO, (c) Fe_3_O_4_-GO. Figure S2: Removal rates of (a) Zn, (b) Cu, (c) Pb, (d) Cd, and (e) Cr by different domesticated generations of strains. G1–G4 denote the 1st to 4th domesticated generations. Different letters indicate significant differences between generations (*p* < 0.05). Figure S3: Removal rates of (a) Zn, (b) Cu, (c) Pb, (d) Cd, and (e) Cr over time for Group 1 (modified anode + bacteria + electricity + Fe_3_O_4_-GO) and Group 3 (modified anode + bacteria + electricity). “d” = days. Figure S4: Removal rates of (a) Zn, (b) Cu, (c) Pb, (d) Cd, and (e) Cr over time for Group 1 (modified anode + bacteria + electricity + Fe_3_O_4_-GO) and Group 4 (modified anode + bacteria + Fe_3_O_4_-GO). “d” = days. Figure S5: Removal rates of (a) Zn, (b) Cu, (c) Pb, (d) Cd, and (e) Cr over time for Group 1 (modified anode + bacteria + electricity + Fe_3_O_4_-GO) and Group 5 (unmodified anode + bacteria + electricity + Fe_3_O_4_-GO). “d” = days.

## Abbreviations

The following abbreviations are used in this manuscript:

GO	Graphene Oxide
BES	Bioelectrochemical System
EET	Extracellular electron transfer
SMFCs	Sediment microbial fuel cells
AAMCC	Aluminum alloy mesh/carbon cloth composite electrode
*A. ferrooxidans*	*Acidithiobacillus ferrooxidans*
PTEs	potentially toxic elements
ORP	Oxidation–Reduction potential
