# Supplementary material for: Bioremediation of High-Concentration Heavy Metal-Contaminated Soil by Combined Use of Acidithiobacillus ferrooxidans and Fe3O4–GO Anodes
Source: Toxics. 2025 Nov 6;13(11):959. doi: 10.3390/toxics13110959 (PMC12656165; doi:10.3390/toxics13110959)
Supplement: Supplementary file 1 [file toxics-13-00959-s001.zip › toxics-3898609-supplementary.pdf]

supplementary material

Table S1. Basic Properties of the Test Soil Sample.

	Soil Sample	GB15618—2018	GB36600—2018
pH	7.85	＞7.5	/
Conductivity（mS/cm)	2.3	/	/
Zn Mass Ratio/（mg/kg）	17501	300	400
Cu Mass Ratio /（mg/kg）	4986	100	2000
Pb Mass Ratio /（mg/kg）	5559	170	400
Cd Mass Ratio /（mg/kg）	38	0.6	20
Cr Mass Ratio /（mg/kg）	674	250	3
As Mass Ratio /（mg/kg）	149	25	20

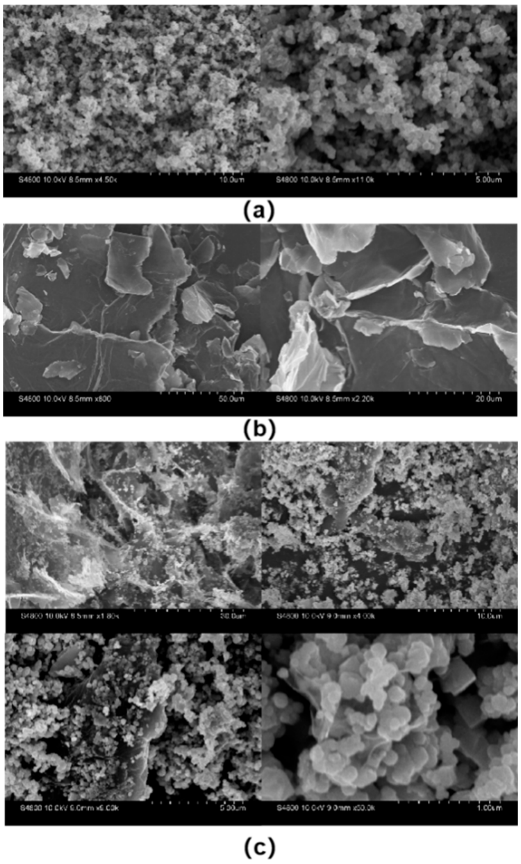

Figure S1. Electron microscopy of (a) Fe3O4, (b) GO, (c) Fe3O4-GO.

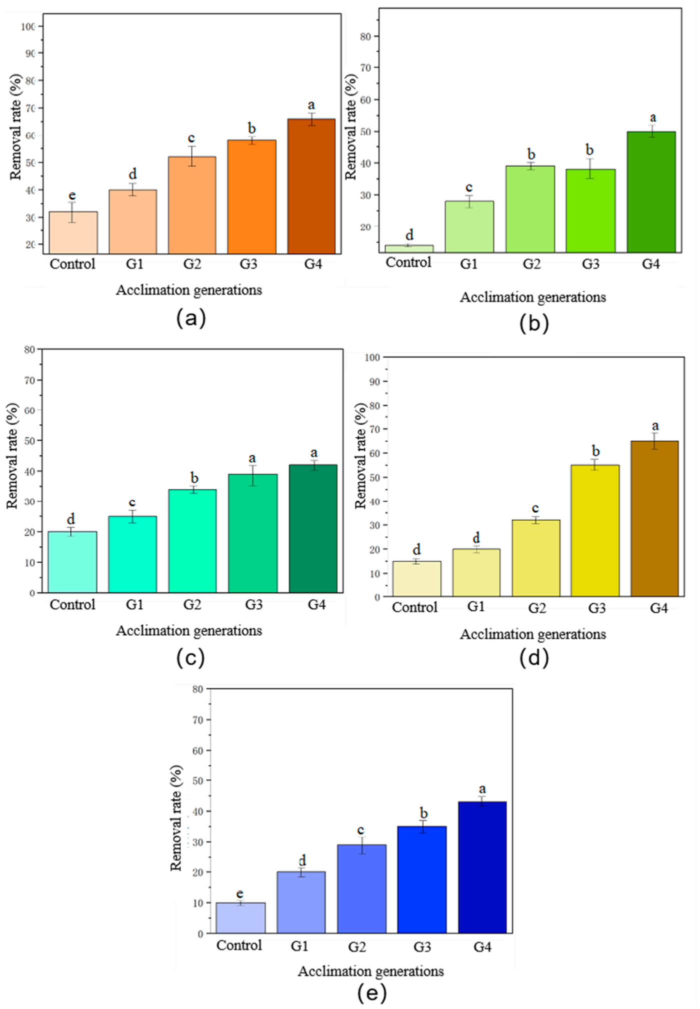

Figure S2. Removal rates of (a) Zn, (b) Cu, (c) Pb, (d) Cd, and (e) Cr by different domesticated generations of strains. G1–G4 denote the 1st to 4th domesticated generations. Different letters indicate significant differences between generations ($p < 0.05$).

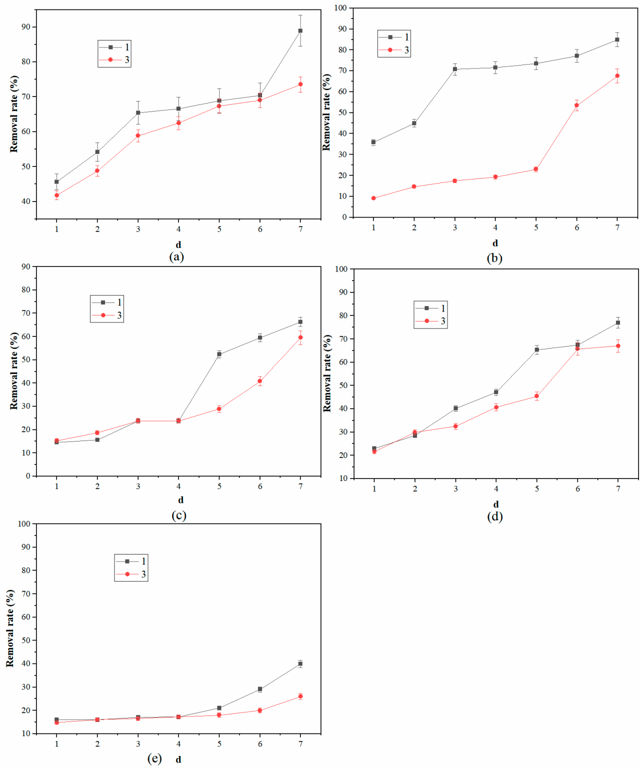

Figure S3. Removal rates of (a) Zn, (b) Cu, (c) Pb, (d) Cd, and (e) Cr over time for Group 1 (modified anode + bacteria + electricity + Fe_3O_4-GO) and Group 3 (modified anode + bacteria + electricity). "d" = days.

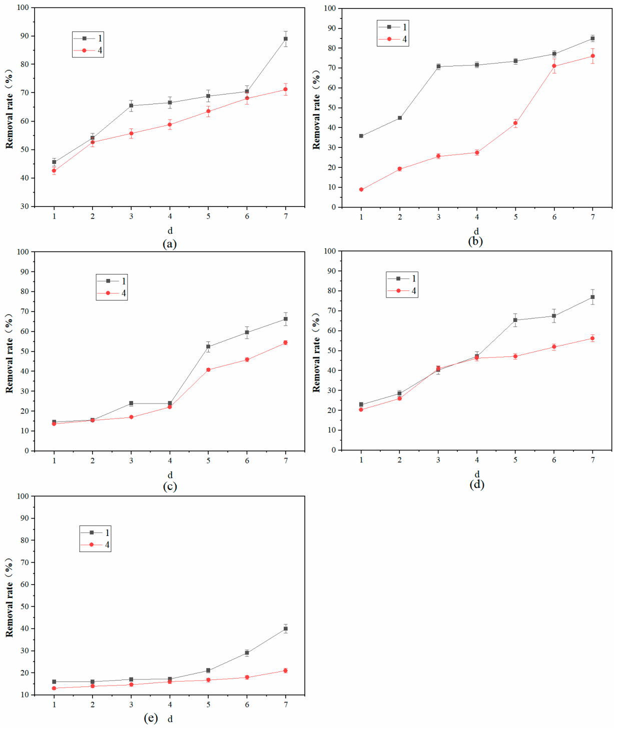

Figure S4. Removal rates of (a) Zn, (b) Cu, (c) Pb, (d) Cd, and (e) Cr over time for Group 1 (modified anode + bacteria + electricity + Fe_3O_4-GO) and Group 4 (modified anode + bacteria + Fe_3O_4-GO). "d" = days.

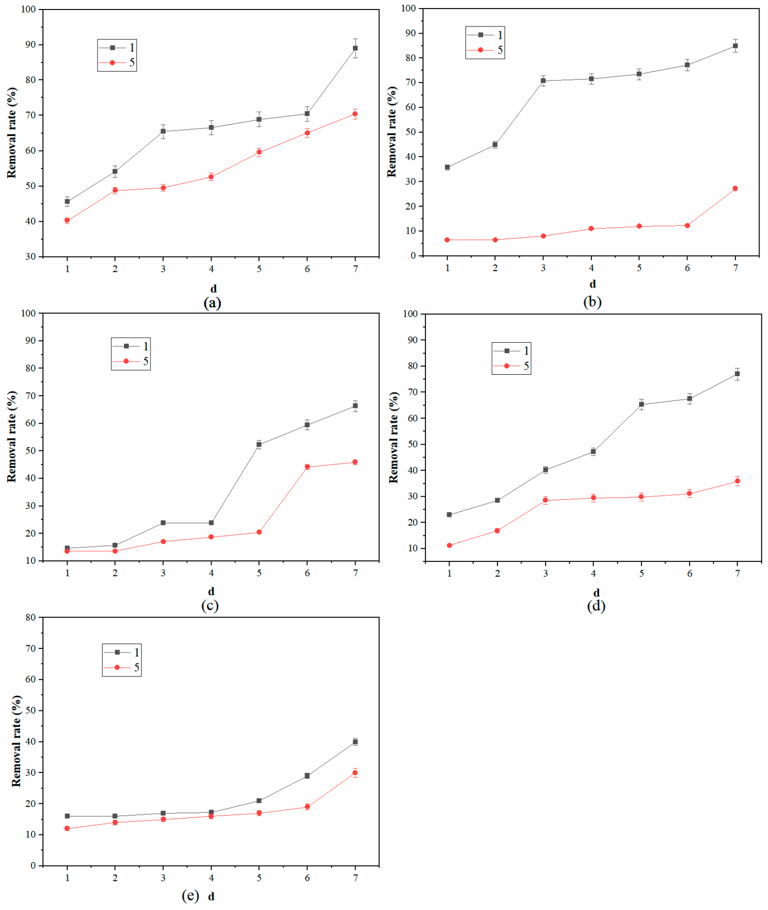

Figure S5. Removal rates of (a) Zn, (b) Cu, (c) Pb, (d) Cd, and (e) Cr over time for Group 1 (modified anode + bacteria + electricity + Fe_3O_4-GO) and Group 5 (unmodified anode + bacteria + electricity + Fe_3O_4-GO). “d” = days.
